# Prevalence of oral lesions among Saudi dental patients

**DOI:** 10.4103/0256-4947.55166

**Published:** 2009

**Authors:** Azizah Al-Mobeeriek, Abdullah M. AlDosari

**Affiliations:** From the Department of Maxillofacial Surgery and Diagnostic Sciences, College of Dentistry, King Saud University, Riyadh, Saudi Arabia

## Abstract

**BACKGROUND AND OBJECTIVES:**

Few studies have been conducted in the Saudi population on oral mucosal lesions. The purpose of this study was to evaluate the type and extent of oral lesions in a study among dental patients at a college of dentistry in Saudi Arabia.

**PATIENTS AND METHODS:**

Over a 3-year period, 2552 dental outpatients were interviewed and investigated clinically for the presence of oral mucosal conditions. A thorough oral clinical examination was performed, including a radiographic examination. The diagnosis was confirmed histopathologically when necessary.

**RESULTS:**

Of 383 (15.0%) patients found to have oral mucosal lesions, females constituted 57.7% (n=221) and males 42.3% (n=162). The age range of the patients was between 15 to 73 years with a mean age of 38.2 years. The most commonly affected age group was 31 to 40 years, which comprised 21.4% (n=82) of all affected individuals. The least affected age group were individuals older than 61 years. The most common lesion was Fordyce granules (3.8%; n=98), followed by leukoedema (3.4%; n=86) and traumatic lesions (ulcer, erosion) in 1.9% (n=48). Tongue abnormalities were present in 4.0% (n=101) of all oral conditions observed, ranging from 1.4% (n=36) for fissured tongue to 0.1% (n=2) for bifid tongue. Other findings detected were torous platinus (1.3%; n=34), mandibular tori (0.1%; n=2) aphthous ulcer (0.4%; n=10), herpes simplex (0.3%; n=7), frictional hyperkeratosis (0.9%; n=23), melanosis (0.6%; n=14), lichen planus (0.3%; n=9) and nicotinic stomatitis (0.5%; n=13).

**CONCLUSION:**

The findings of this study provide information on the types and prevalence of oral lesions among Saudi dental patients. This provides baseline data for future studies about the prevalence of oral lesions in the general population.

Few studies have been conducted in the Saudi population on oral mucosal lesions. An 8-year retrospective study using biopsied specimens found that focal fibrous hyperplasia (FFH), pyogenic granuloma (PG), peripheral giant cell granuloma (PGCG) and peripheral ossifying fibroma (POF) constitute 9% of all biopsied lesions.^1^ A 5-year (1985-1990) survey in Saudi Arabia indicated that 64.6% of biopsied lesions were benign tumors, hyperplasia and granulomas, 20.3% were cysts and cyst-like lesions and 4.8% were other mucous membrane lesions.^2^

The prevalence of leukoplakia was studied in relationship to tobacco habits and was found to be 11.4% in the Gizan area of Saudi Arabia.^3^,^4^ The oral cavity was ranked as one of the most common ten malignancies from 1975-2006.^5^ The tongue was the most affected site (30.1%), mostly on the lateral borders of the anterior two-thirds, followed by the oropharynx, lips, cheeks, gingiva and floor of the mouth, palate and salivary gland.^6^ Oral health survey data are essential for proper health planning programs. In addition, most of the conducted studies were of tumors or ulcers and did not include all oral lesions in adult populations. The pattern of disease is also changing due to increasing awareness, changes in lifestyle and increasing interest in oral health. In Saudi Arabia there is a great need for clinical studies to establish baseline data on the prevalence of oral lesions. The aim of this study was to determine the type and the prevalence of oral lesions among patients seeking dental care.

## PATIENTS AND METHODS

This study was carried out on adult Saudi dental outpatients who attended the Oral Diagnostic Clinic at the College of Dentistry, King Saud University, Riyadh, Saudi Arabia, for an oral examination and a dental treatment plan. The study sample included adult subjects who were older than 15 years of age. The college is an open public facility and referral hospital. A total of 2552 patients were interviewed and clinically investigated for the presence of oral lesions from June 2002- to December 2005. History taking and a thorough oral clinical examination was performed, including a radiographic examination. The type and distribution of the oral mucosal conditions was recorded. An interview was conducted to collect information. The examination was performed by a single examiner. Data were statistically analyzed using SPSS (SPSS, Inc. Chicago, IL).

## RESULTS

**Figure 1:**
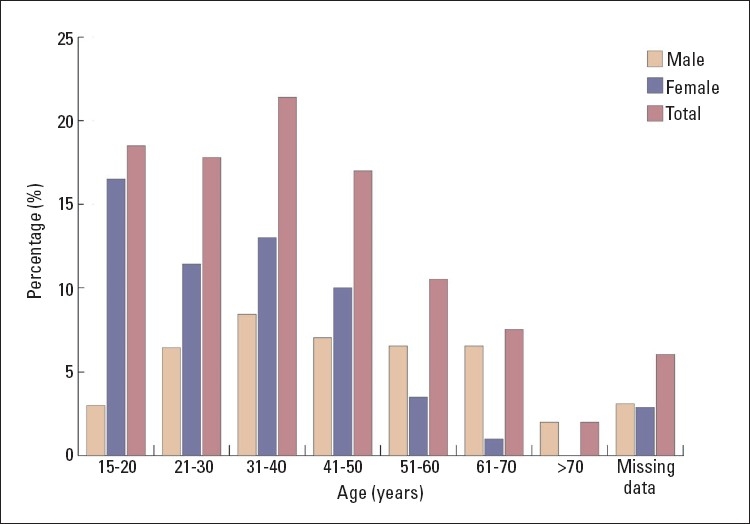
Prevalence of oral conditions by age range and sex (n=383).

Among the 2552 patients, only 383 patients (15.0%) had oral lesions. Females constituted 57.7% (n=221) and males 42.3% (n=162). The age range of the patients was between 15 to 73 years. The mean age of the sample group was 38.2 years. The mean age for females was 33.0 years and 47.1 years for males. Of the total sample, the most commonly affected age was between 31 to 40 years (21.4%), followed by 15 to 20 years (18.5%), and 21 to 30 years (17.8%) (Figure 1). Twenty-four patients (0.9%) admitted smoking habits and 196 patients (7.7%) had a systemic disease, but there were no differences between smokers and non-smokers, or between healthy people and those with systemic diseases.

Oral lesions were more prevalent among females than males (Table 1). Fordyce granules were observed in 3.84% (n=98) on the buccal and labial mucosa, and were significantly more common in females. Females showed a higher prevalence than males, accounting for two-thirds of the affected individuals (n=24), but the difference was not statistically significant. Both torous platinus and mandibular tori wre more common in females, but the difference was not statistically significant. Other lesions were significantly more common among females: aphthous ulcer and herpes simplex. On the other hand, frictional hyperkeratosis was more common among males than females, but the difference was not statistically significant. Other pathologies detected were excessive melanin pigmentation (melanosis). Females showed a higher incidence than males, but the difference was not statistically significant. Both lichen planis and nicotinic stomatitis were significantly higher among males.

**Table 1:** Distribution of oral conditions in Saudi dental patients (n=2552).

Type	Males		Females		Total	
	No.	% of total sample	No.	% of total sample	No.	% of total sample
Fordyce granules^a^	30	1.18	68	2.67	98	3.84
Leukoedema^b^	53	2.08	33	1.29	86	3.37
Traumatic lesions (ulcers, erosions)	14	0.55	34	1.33*	48	1.88
Torous platinus	0	0	34	1.33	34	1.33
Frictional hyperkeratosis	14	0.55	9	0.35	23	0.90
Melanosis	5	0.195	9	0.35	14	0.55
Nicotinic stomatitis^a^	11	0.43	2	0.08	13	0.51
Aphthae^a^	0	0	10	0.39	10	0.39
Lichen planus	7	0.27	2	0.08	9	0.35
Herpes simplex^c^	0	0	7	0.27	7	0.27
Mandibular tori	0	0	2	0.08	2	0.08
Non-nicotinic stomatitis	2	0.08	2	0.08	4	0.16
Focal fibrous hyperplasia	2	0.08	3	0.12	5	0.195

Tongue lesions						
Fissured tongue	12	0.47	24	0.94	36	1.41
Bifid tongue	0	0	2	0.08	2	0.08
Glossitis	0	0	3	0.12	3	0.12
Scalloped tongue	0	0	8	0.31	8	0.31
Lingual varcosis	2	0.08	8	0.31	10	0.39
Geographic tongue	7	0.27	6	0.24	13	0.51
Hairy tongue^c^	10	0.39	4	0.16	14	0.55
Tongue tie	2	0.08	12	0.47	15*	0.59
Total tongue lesions	33	1.3	67	2.6	101	3.96

**Total**	171		282		454	

* One case missing sex;

a *P*<.005,

b *P*=.0001,

c *P*<.05 males vs. females

Tongue abnormalities were present in 3.96% (n=101) of total sample and in 26.4% of all oral conditions observed. The prevalence of tongue lesions was higher among females than males, but the difference was not statistically significant. the most common tongue condition was fissured tongue, constituting about 35.6% of all tongue conditions. it is also ranked fourth most common lesion in our population. Hairy tongue was present in 0.55% of the sample (n=14) and was significantly more common in males than in females. Tongue tie was also significanlty more common in females than males. Other tongue lesions were lingual varcosis. Females showed a higher incidence than males, but the difference was no statistically significant. Both bifid tonge and glossitis had a low occurrence and were seen manily among females.

## DISCUSSION

Oral soft tissue lesions present a significant health problem with a considerable morbidity. Despite its importance, there are few reports on its prevalence among the Saudi population and its association with oral habits, when compared to dental caries and periodontal diseases.

In accordance with others,^1^,^2^ our results showed a higher prevalence of oral mucosal lesions among females (57.7%) and young adults (31-40 years) (21.4%). Other reports, however, indicated that oral lesions tend to increase with age in association with tobacco consumption and denture use.^7^,^8^ The age of the patient is crucial in patient assessment, treatment planning and health education.

The present study suggests the distribution pattern of oral diseases in Saudi Arabia is similar to other countries. Benign lesions are more common in the young and females while cancerous and precancerous lesions are more common in the elderly.^9^,^10^ Along with other investigations,^1^–^6^,^11^ it may also shed some light on the pattern of oral diseases among the Saudi population. Reports from different regions of Saudi Arabia have shown that cancer and precancerous lesions are more common in elderly males^3^–^6^,^11^ in contrast to benign lesions, which are more common in females at a younger age.^1^,^2^

As in the report of Kovac-Kovacic and Skaleric in Slovenia,^10^ Fordyce granules were the most common oral condition and had a female predilection (10%). This lesion was not as common among Amazonian Indians (3.8%).^12^ Leukoedema was found to have a high prevalence among our study population. Similar findings have been reported among Kenyans (26%), the Swedish population (49.07%) and in the institutionalized elderly in South Africa (24.4%).^13^–^15^ In contrast, leukoedema was not reported as one of the common soft tissue conditions among elderly Chinese.^16^ As in other studies, males were significantly more affected than females.^11^,^17^ Studies have demonstrated a positive association between smoking, tobacco use and leukoedema with a decline the size of the lesion after tobacco cessation.^17^–^20^ Though not clear in our results, this may in general explain the male predominance of this condition.

Traumatic ulcer was the third most common soft tissue lesion. Females were affected more than males. It was also found to be one of the common soft tissue lesions in Spain, Italy, and Chile elderly and in the institutionalized elderly in Denmark.^21^–^24^ The main reason among the elderly was poorly constructed dentures.^23^–^25^

As in the to Kenya^13^ and in elderly Malaysians,^26^ frictional hyperkeratosis ranked as one of the most common oral mucosal lesions. This finding supports the results from the United States of America,^27^ China^28^ and Bangladesh,^29^ where keratotic lesions are a common clinical presentation possibly due to population differences, particularly in term of tobacco habits.

Consistent with data from China,^28^ tongue conditions were a frequent observation in our study, comprising almost one-third of the mucosal lesions. Fissured tongue ranked fourth among oral abnormalities and was the most common tongue conditions. Worldwide, fissured tongue occurrence varies, but remains a common tongue condition ranging from 28% among elderly Thai,^30^ 27.3% among Amazonians,^12^ 21.1% among Slovenians^10^ and 5.2% in Turkey.^31^ Fissured tongue was also one of the common tongue conditions, constituting 45.7% in Jordan.^32^

The findings of this study possibly provide important and missing information about the types and prevalence of oral lesions among Saudi dental patients and can serve as baseline data for future studies on the prevalence of different oral lesions in the general population.
